# Integrated circuits based on conjugated polymer monolayer

**DOI:** 10.1038/s41467-017-02805-5

**Published:** 2018-01-31

**Authors:** Mengmeng Li, Deepthi Kamath Mangalore, Jingbo Zhao, Joshua H. Carpenter, Hongping Yan, Harald Ade, He Yan, Klaus Müllen, Paul W. M. Blom, Wojciech Pisula, Dago M. de Leeuw, Kamal Asadi

**Affiliations:** 1 Max Planck Institute for Polymer Research, Ackermannweg 10, Mainz, 55128 Germany; 2 Department of Chemistry, The Hong Kong University of Science and Technology, Clear Water Bay, Kowloon, Hong Kong; 3 Department of Physics and ORaCEL, North Carolina State University, Raleigh, 27695 NC USA; 4 SLAC National Accelerator Laboratory, Menlo Park, 94025 CA USA; 5 Department of Molecular Physics, Faculty of Chemistry, Lodz University of Technology, Zeromskiego 116, Lodz, 90-924 Poland; 6 Faculty of Aerospace Engineering, Delft University of Technology, Kluyverweg 1, Delft, 2629 HS The Netherlands; 7 Present Address: Molecular Materials and Nanosystems, Institute for Complex Molecular Systems, Eindhoven University of Technology, P.O. Box 513, Eindhoven, 5600 MB The Netherlands

## Abstract

It is still a great challenge to fabricate conjugated polymer monolayer field-effect transistors (PoM-FETs) due to intricate crystallization and film formation of conjugated polymers. Here we demonstrate PoM-FETs based on a single monolayer of a conjugated polymer. The resulting PoM-FETs are highly reproducible and exhibit charge carrier mobilities reaching 3 cm^2^ V^−1^ s^−1^. The high performance is attributed to the strong interactions of the polymer chains present already in solution leading to pronounced edge-on packing and well-defined microstructure in the monolayer. The high reproducibility enables the integration of discrete unipolar PoM-FETs into inverters and ring oscillators. Real logic functionality has been demonstrated by constructing a 15-bit code generator in which hundreds of self-assembled PoM-FETs are addressed simultaneously. Our results provide the state-of-the-art example of integrated circuits based on a conjugated polymer monolayer, opening prospective pathways for bottom-up organic electronics.

## Abstract (web summary)

Polymer monolayer field-effect transistors hold promise for faster circuits, but their performance is currently limited by the polymer packing disorder. Li et al. pre-aggregate polymers in a solution to achieve high carrier mobility of 3 cm^2^ V^−1^s^−1^ in monolayers and utilize them in integrated circuits.

## Introduction

The few first layers of organic semiconductors adjacent to the gate dielectric dominate the charge carrier transport in organic field-effect transistors (OFETs)^1,2^. OFETs based on a single-molecular layer (monolayer) of semiconductors are of particular interest. The monolayer-thick transistors, hereafter called monolayer transistors, provide a near-ideal platform for the investigation of fundamental transport mechanisms, due to the two-dimensional nature of the monolayer and therefore confinement of the charge carriers and their pathways to two dimensions^3^. Monolayer transistors hold great potentials in applications for chemical and biological sensors due to accessibility of the transistor channel and therefore exhibiting higher sensitivity, faster response/recovery rate, better selectivity, and lower detection limits^4–9^.

Considerable progress has been made on the organic monolayer transistors based on conjugated small molecules. For instance, thermal evaporation in high vacuum allowed for the fabrication of monolayers of pentacene^10^, oligothiophene^11^, and their derivatives^8^ with the field-effect mobility of around 10^−2^ cm^2^ V^−1^ s^−1^. Solution processing by drop-casting was also capable of depositing the monolayer-thick ultrathin films or even single crystals, and the reported mobility ranged from 10^−2^ to 1 cm^2^ V^−1^ s^−1^^5,12–14^. Self-assembly is another efficient bottom-up route to downscale the semiconductor layer into a monolayer^15–19^. By chemically modifying the *π*–conjugated semiconducting core with an anchoring group capable of covalent binding to the dielectric surface, self-assembled-monolayer field-effect transistors (SAMFETs) could be achieved with the mobility of 0.01–0.04 cm^2^ V^−1^ s^−1^, enabling realization of functional integrated circuits^20–23^.

Semiconducting conjugated polymers have also been widely investigated due to their flexibility and solution processability. However, it is still a great challenge to obtain high charge carrier mobility in a conjugated polymer monolayer field-effect transistor (PoM-FET). The deposition of polymer monolayer with the thickness of 2–4 nm has been realized through spin-coating, Langmuir–Blodgett, Langmuir–Schäfer, and bar-coating techniques, but the resultant PoM-FETs generally exhibited a field-effect mobility on the order of 10^−2^ cm^2^ V^−1^ s^−1^ or even lower (Supplementary Table 1)^24–31^. Recently, the bar-coating process has been employed to fabricate poly{[N,N′-bis(2-octyldodecyl)-naphthalene-1,4,5,8-bis(dicarboximide)-2,6-diyl]-alt-5,5′-(2,2′-bithiophene)} (P(NDI2OD-T2)) with aligned nanofiber network along the processing direction^32^. In spite of little long-rang in-plane alignment of the polymer backbones, PoM-FETs showed an electron mobility of 0.14 cm^2^ V^−1^ s^−1^, one order of magnitude higher than the previous reports. The performance was further improved on the basis of poly[[2,5-bis(2-octyldodecyl)-2,3,5,6-tetrahydro-3,6-dioxopyrrolo[3,4-c]pyrrole-1,4-diyl]-alt-[[2,2′-(2,5-thiophene)bis-thieno(3,2-b)thiophene]-5,5′-diyl]] (DPPT-TT) using the same deposition technique leading to a field-effect mobility of around 1.1 cm^2^ V^−1^ s^−1^^33^. The thickness-dependent mobility for the DPPT-TT PoM-FETs implied a relatively low molecular ordering in the first polymeric monolayers which play a major role in charge transport. Moreover, reported threshold voltage (*V*_T_) of tens of Volts is large for practical applications. Therefore, high-mobility PoM-FETs with reasonable operating conditions are still in high demand for bottom-up organic electronics.

Here we present an integrated circuit of hundreds of PoM-FETs based on the solution processing of a difluorobenzothiadiazole-oligothiophene copolymer (PffBT4T-2DT) into a well-ordered monolayer. High molecular weight polymers are capable of inducing higher degree of molecular order and consequently facilitating charge transport^34–41^. The synthetic strategy of PffBT4T-2DT is optimized^42^ to increase the molecular weight compared to the previous works^43^. The improved molecular weight lead to the formation of well-defined nanofibrillar microstructures due to the strong long-range *π*−*π* interactions between the chains already present in solution, and an obvious edge-on orientation is directly determined for monolayer-thick polymer film by grazing incident wide-angle X-ray scattering (GIWAXS), indicating high degree of molecular ordering that is persistent down to the monolayer of polymer. Direct observation of strong long-range in-plane order of the polymer backbones in the monolayer-thick film has not been reported so far^32^. Pronounced organization of polymer chains greatly facilitates charge carrier transport within the polymer monolayer, resulting in the field-effect mobility up to 3 cm^2^ V^−1^ s^−1^. In combination with improved FET fabrication process and contact engineering, the high molecular order results in high mobility, large current modulation and high reproducibility of PffBT4T-2DT PoM-FETs, which further allow combination of the PoM-FETs into inverters to form unipolar logic gates. Ring oscillators are realized due to the small parameter spread of the individual inverters. We further demonstrate the state-of-the-art example of an integrated circuit, IC, based on a conjugated polymer monolayer by constructing a 15-bit code generator, suggesting the great potential for bottom-up organic electronics.

## Results

### Controllable growth of conjugated polymer monolayer

**Fig. 1:**
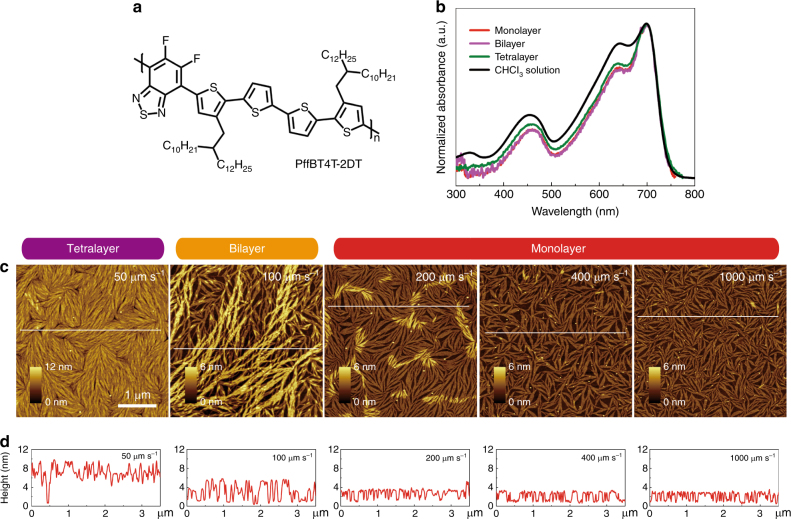
Optical properties and microstructures of PffBT4T-2DT mono- to multilayers. **a** Chemical structure of PffBT4T-2DT. **b** UV–Vis absorption spectra of PffBT4T-2DT as thin films and in solution of chloroform (0.025 mg mL^−1^, 25 °C). Thin films are deposited on quartz wafers by dip-coating from 0.5 mg mL^−1^ chloroform solution at room temperature. **c** AFM images of PffBT4T-2DT ultrathin films from multilayers down to monolayer obtained at different dip-coating speeds (50, 100, 200, 400, and 1000 μm s^−1^) from 0.5 mg mL^−1^ chloroform solution. All AFM images have the same scale *x*, *y*, and *z* bars except the height scale of the tetralayer. **d** The corresponding height profiles along the indicated lines in **c**

A donor–acceptor conjugated polymer, PffBT4T-2DT (Fig. 1a), was deposited on heavily doped silicon with thermally grown SiO_2_ (SiO_2_/Si) from a 0.5 mg mL^−1^ chloroform solution by a well-controlled dip-coating process with monolayer precision^43–46^. To engineer the microstructure, as will be explained later, choosing a solvent that ensures sufficient solubility and effective pre-aggregation of polymer in solution is of vital importance. It has been shown that dissolution of PffBT4T-2DT in a solvent with an inferior content of aggregates, such as trichlorobenzene, leads to the formation of thin film with obvious molecular disorder and face-on orientation, which results in inferior charge carrier mobility^47^. The root-mean-square roughness of the substrate is around 0.2 nm, which has no impact on the polymer self-assembly. Tapping mode atomic force microscopy (AFM) and grazing incident wide-angle X-ray scattering (GIWAXS) are performed to analyze the film microstructure, molecular packing and thickness. The dip-coating speed plays a crucial role in the microstructure formation and the final film thickness (Supplementary Fig. 1). At 50 μm s^−1^, the layer thickness is 9.8 nm, which is equivalent to four molecular layers, hence referred to as tetralayer (Fig. 1c, d). By increasing the dip-coating speed to 100 μm s^−1^, the film thickness decreases to 5.0 nm (a bilayer). By further increase in the dip-coating speed to 200 μm s^−1^, the film thickness is reduced to 2.4 nm which corresponds to the thickness of a PffBT4T-2DT chain with edge-on orientation. The monolayer thickness is further confirmed by the interlayer distance and thickness found in the GIWAXS analysis, as will be discussed. The PffBT4T-2DT monolayer consists of randomly oriented nanofibers with a median length of 724 ± 217 nm and width of 73 ± 26 nm. The monolayer coverage is nearly 80%, calculated from the AFM height images. The lateral fiber dimensions (width and length) of the monolayer are identical to that of the bi- and tetralayer, as shown in Supplementary Fig. 2. With a coating speed of 200 μm s^−1^, the nuclei of the second layer start to grow on top of the first monolayer (Fig. 1c), but its growth is minimal and its coverage is < 7%, hence the film is considered as a monolayer. When the dip-coating speed is further increased, both fiber dimension and monolayer coverage are remarkably reduced. For instance, at 1000 μm s^−1^ the nanofibers of the polymer monolayer are 389 ± 138 nm in length and 48 ± 18 nm in width, with the coverage of 62%.

**Fig. 2:**
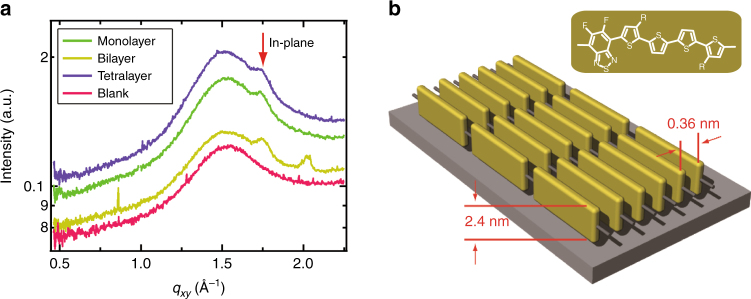
Molecular order of PffBT4T-2DT monolayer. **a** In-plane GIWAXS line profiles. Profiles are offset for clarity. The in-plane (010) *π*–*π* peaks at *q* = 1.75 Å^−1^ are indicated by red arrow. A bare substrate (blank) is also measured for reference (The feature at *q* ≈ 2 Å^−1^ is parasitic scatter from Si dust). **b** Schematic illustration of a single nanofiber of PffBT4T-2DT monolayer with an edge-on orientation, in which the brown bricks represent the monomer units of PffBT4T-2DT. The arrangement of backbones relative to each other is idealized. In reality, the monomers might not be in registry

The microstructure formation can be qualitatively explained as follows. Observation of the identical UV–Vis spectra for the polymer film and chloroform solution (Fig. 1b), and the insensitivity of the molecular packing of the film on the coating speed (see below, Fig. 2a) indicate the presence of pre-aggregation in the polymer solution^47–49^. Formation of pre-aggregates is proven by temperature dependent UV–Vis spectra of the polymer solution, as shown in Supplementary Fig. 3. We note that the size of the pre-aggregate is small enough that it cannot be detected by dynamic light scattering (DLS). The pre-aggregates act as nucleation center for the growth of the polymer layer. The higher solute concentration near the contact line increases the local concentration of PffBT4T-2DT pre-aggregates, which finally leads to the formation of nucleation centers at the contact line. The fingering instability of the contact line induces a well-defined fibrillary microstructure^50–53^. We note that inks with high content of aggregates were capable of fine tuning film thickness down to the formation of an interconnected monomolecular layer^32^.

### Molecular order in polymer monolayer

GIWAXS is used to probe the in-plane organization and qualitative orientational texture of the PffBT4T-2DT mono- and multilayers. The corresponding 1D profiles are shown in Fig. 2a. Several scattering peaks arising from the interlayer spacing of 2.4 nm for the PffBT4T-2DT tetralayer are visible in the out-of-plane profile at *q* = 0.26, 0.51, and 0.76 Å^−1^ (Supplementary Fig. 4), in agreement with the monolayer size of edge-on oriented chains. More importantly, in the in-plane profiles the (010) *π*–*π* peaks at *q* = 1.75 Å^−1^ (indicated by the red arrow in Fig. 2a), corresponding to a *π*-stacking distance of 0.36 nm, are apparent down to the monolayer. Identical results are obtained at a different beamline (Advanced Light Source, ALS, Supplementary Fig. 5). The characteristic in- and out-of-plane positions of the *π*-stacking and interlayer peaks, respectively, and their complementary intensities indicate highly ordered polymer films with pronounced edge-on orientation of the chains from multilayers down to a monolayer with structural organization as schematically shown in Fig. 2b.

To quantitatively investigate the in-plane long-range order of the monolayer films, (010) coherence length is calculated based on the Scherrer equation from peak fits to the in-plane GIWAXS data shown in Fig. 2a^54^. Films coated at speeds of 50 µm s^−1^, 100 µm s^−1^, and 200 µm s^−1^ exhibit coherence lengths of 7.7 ± 0.2 nm, 8.0 ± 0.3 nm, and 8.2 ± 0.3 nm, respectively. The marginal increase in the coherence length indicates that the coating speed has a negligible impact on the degree of ordering in the *π*-stacking direction of the polymer films down to a monolayer. Highly ordered, preferential orientation packing of the polymer in monolayer insensitive to the coating speed, further corroborates the importance of pre-aggregation in solution.

### Device performance of polymer monolayer transistor

Field-effect transistors with bottom-contact bottom-gate (BCBG) configuration are used to characterize charge carrier transport in the PffBT4T-2DT monolayer. We note that using top-contact bottom-gate geometry (TCBG) for transistors, as shown in Supplementary Fig. 9, leads to inferior FET performance in comparison with BCBG. Heavily doped silicon wafers with 200-nm-thick thermally grown SiO_2_ dielectric are utilized as substrate. Source and drain (S/D) electrodes are patterned using conventional lithography prior to deposition of the polymer monolayer. To lower the contact resistance^55,56^, the Au electrodes are functionalized by 2,3,4,5,6-pentafluorothiophenol (PFBT) self-assembled monolayer (SAM) before the deposition of the polymeric monolayer (Supplementary Fig. 8)^57^. The SiO_2_ dielectric remains unmodified to ensure sufficient wettability of the substrate during the deposition polymer monolayer deposition. To achieve a high PffBT4T-2DT monolayer coverage and large fiber dimensions, a dip-coating speed of 200 μm s^−1^ is used for the fabrication of the transistor (Fig. 1). We find that the PFBT modification significantly decreases the surface tension of Au electrodes (Supplementary Fig. 8), which plays a crucial role in the morphology of polymeric monolayer (Supplementary Fig. 13). However, the morphology and molecular order of the polymeric monolayer in the middle of the conducting channel are not affected by the presence of the Au or Au/PFBT electrodes (Supplementary Fig. 6). Subsequently, the polymer monolayer is annealed at 100 °C for 30 min to remove the residual solvent without affecting the morphology and molecular organization of PffBT4T-2DT thin films^43,48^.

**Fig. 3:**
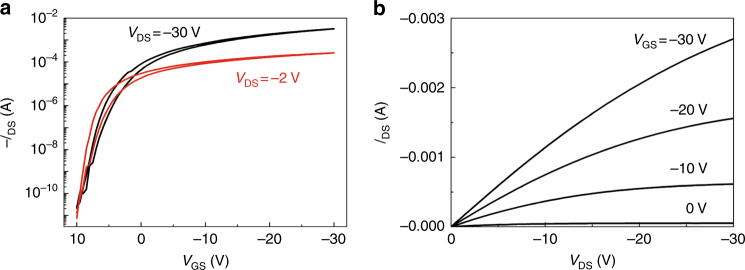
Device performance of PffBT4T-2DT PoM-FETs. **a** Transfer and **b** output characteristics of PffBT4T-2DT PoM-FET with co-centric ring geometry. The channel length and width are 10 and 2500 μm, respectively. The drain voltages used in (**a**) are −2 and −30 V for the measurement in the linear and saturation regimes, respectively

PoM-FET transfer and output characteristics of PffBT4T-2DT are shown in Fig. 3a, b exhibiting a typical linear/saturation behavior. The transfer plots are characterized in both linear and saturation regimes at drain voltages (*V*_DS_) of −2 and −30 V. The PoM-FET exhibits hole field-effect mobilities of *μ*_lin_ = 1.02 cm^2^ V^−1^ s^−1^ and *μ*_sat_ = 2.08 cm^2^ V^−1^ s^−1^ extracted from the transfer curves. The mobility is thermally activated with an activation energy of about 90 meV (Supplementary Fig. 11). Near-ideal output plots observed in Fig. 3b, especially at low *V*_DS_, indicate good contact between Au electrodes and the polymeric monolayer due to PFBT modification^55^. We note that the Au modification is crucial for a good transistor performance since the unmodified Au electrodes lead to lower degree of polymer ordering at the contact, higher contact resistance and lower current (Supplementary Fig. 12,13). The PoM-FET performance is similar to the that of the FETs made of PffBT4T-2DT bulk film^48^. Both PffBT4T-2DT bi- and tetralayer show also identical saturation mobilities to the monolayer (Supplementary Fig. 14), confirming that the charge carriers are mainly transported in the first monolayer adjacent to the dielectric layer^2,58^. The similarity in performance can be attributed to the excellent molecular ordering as indicated by GIWAXS. The higher molecular weight is capable of enhancing the molecular order and creating a denser interconnection of the ordered regions, thereby improving the transistor performance^34–41^. GIWAXS for tetralayers of a low molecular weight (*M*_n_ = 23.2 kg mol^−1^)^43^ and high molecular weight (*M*_n_ = 47.3 kg mol^−1^) PffBT4T-2DT is performed. A comparison of the in-plane GIWAXS profiles are provided in Supplementary Fig. 7. It is clear that the *π*−*π* stacking peak at *q* = 1.75 Å^−1^ disappears for the low molecular weight PffBT4T-2DT, indicating the presence of much lower molecular order. In contrast, the *π*−*π* stacking peak for high molecular weight PffBT4T-2DT is visible and persist down to the monolayer, Fig.  2a.

To show robustness and high reproducibility of the PffBT4T-2DT PoM-FETs, transistors with different S/D electrode geometries i.e., co-centric rings, interdigitated, and linear structures are fabricated. In excess of 80 discrete PoM-FETs are measured. The results are summarized in Supplementary Table 2 and Supplementary Fig. 10. Threshold voltages (*V*_T_) and on/off ratios (*I*_on_/*I*_off_) are similar irrespective of the electrode geometries. Moreover, nearly identical values of 0.90 ± 0.28 cm^2^ V^−1^ s^−1^ and 1.31 ± 0.41 cm^2^ V^−1^ s^−1^ are obtained for the linear (*μ*_lin_) and saturation mobility (*μ*_sat_), respectively. The maximum value of *μ*_sat_ reaches as high as 3.02 cm^2^ V^−1^ s^−1^, is a record for FETs based on polymeric monolayers. We note that similar mobility values for both linear and saturation regimes are theoretically expected. Accumulated charge carriers in a FET are confined at the semiconductor/dielectric interface. The charge accumulation layer thickness amounts to 2–3 nm, which is comparable to the thickness of polymer monolayer. Due to the confinement of the charge carriers in the only existing monolayer, the electrical transport in PoM-FETs is two-dimensional. A higher on/off ratio achieved in PoM-FETs can be due to the elimination of the bulk current (Supplementary Fig. 14). Furthermore, the monolayer confines the charge carriers in two dimensions, facilitating the analysis of charge carrier transport^59^.

The isotropy in the charge transport is investigated by fabricating PoM-FETs with various coating directions. It is found that charge carrier mobility is insensitive to alignment of the channels, parallel and perpendicular, with respect to the dip-coating direction. This is indicative of an isotropic semiconductor, i.e., there is no preferential direction for the transport of the charge carriers through the monolayer. Additional evidence for isotropic transport is the identical mobility of co-centric ring devices to interdigitate and linear ones (Supplementary Table 2). Due to the excellent *π*–*π* stacking, the mobility within single fibers can be high. Therefore, when extrinsic effects, such as morphology at the contact are excluded, inter-fiber end-to-end transfer can limit field-effect mobility of the charge carriers.

In an isotropic (or homogenous) semiconductor, the charge carrier mobility does not depend on the transistor channel length for long-channel lengths and is constant^20^.

The contact resistance, particularly in short-channel transistors, negatively influences the mobility of charge carriers leading to lower mobilities. Hence mobilities were obtained from large-channel FETs. Moreover, the increase in mobility with the channel length indicates that charge transport is dominated by structural defects, such as grain boundaries, resulting in an inhomogeneous semiconductor^20^. Channel length scaling for PoM-FETs is shown in Supplementary Fig. 15. For long channels (10–40 μm) mobility is independent of the channel length whereas for short channels (0.5–10.0 μm), both linear and saturation mobilities are reduced with decreasing the channel length, revealing the homogeneous charge transport through the PffBT4T-2DT monolayer. Channel length scaling allows the evaluation of the contact and channel resistance, from which the effective field-effect mobility (*μ*_eff_) can be estimated by using gated circular transfer line method^60^ (CTLM) (Supplementary Fig. 16). The value of 1.3 cm^2^ V^−1^ s^−1^ is obtained for *μ*_eff_, in excellent agreement with the mobility derived from transfer characteristics (Fig. 3 and Supplementary Table 2).

### Integrated circuits based on polymer monolayer

**Fig. 4:**
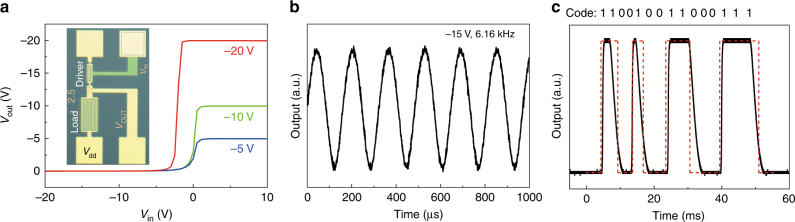
Integrated circuits based on PffBT4T-2DT PoM-FETs. **a** Static input–output characteristics of a unipolar inverter based on PffBT4T-2DT monolayer with *V*_GS_ = 0 V. The supplied voltage, *V*_dd_, is −5 V, −10 V, and −20 V, respectively. The inverter layout is shown as an inset. **b** A PoM-FET 7-stage ring oscillator operating at a frequency of 6.16 kHz with a supply voltage of −15 V. **c** A PoM-FET 15-bit code generator with a bit rate of about 330 bit s^−1^ at *V*_dd_ = −20 V

The narrow parameter spread in discrete PoM-FET, as summarized in Supplementary Table 2, allows realization of an integrated circuit based on a large number of PoM-FETs. Photolithographically defined patterned gate and vertical interconnects, the prerequisites for realization of logic gates and integrated circuits, were adopted from a previously developed 150-mm process technology. Integrated circuits are realized by deposition of an un-patterned PffBT4T-2DT monolayer on the substrate. As shown in Fig. 3a the discrete PoM-FETs are normally on and therefore conducting at zero gate bias. The digital circuit and the logic gates are based on unipolar *V*_GS_ = 0 V inverters, with the source electrode of the load transistor connected to the gate. A photograph of the inverter is shown in the inset of Fig. 4a. The input voltage (*V*_in_) allows the adjustment of the resistance of the driver transistor and essentially controls the output voltage (*V*_out_)^61^. The input–output characteristics of the inverter is presented in Fig. 4a. The input–output characteristics show clear voltage amplification. The corresponding gain depends on the supply voltage (*V*_dd_) with a value of 17.3 at *V*_dd_ = −20 V (Supplementary Fig. 17). Although the gain is lower than the reported record values for polymer thin film transistors^62,63^, it is the only value reported so far for PoM-FET based inverters. Next, a seven-stage ring oscillator (Supplementary Fig. 18) is demonstrated. The output starts oscillating spontaneously at a bias voltage of −5 V and a maximum switching frequency of 6.16 kHz is measured at supplied voltage of −15 V, as shown in Fig. 4b and Supplementary Fig. 19, respectively. The delay time (*t*) can be given by 1/2*Nf*, where *N* and *f* are the number of stages and the switching frequency, respectively^64^. The value of *t* = 11.6 μs is among the lowest reported for ring oscillators based on conjugated polymers and even better than that of carbon nanotube oscillators^64–66^. Real logic functionality is realized by 15-bit code generators. The integrated circuits consist of over 300 PffBT4T-2DT PoM-FETs, an onboard clock generator, a hard-wired memory, a 4-bit counter, a decoder logic, and a load modulator (Supplementary Fig. 20). Figure 4c presents the output characteristics of the 15-bit code generator. A bit rate close to 330 bit s^−1^ is obtained at a supply voltage of −20 V. The circuit performance is comparable to reported state-of-the-art integrated circuits based on bulk organic thin-films, which demonstrates a great potential for the application of the PffBT4T-2DT based PoM-FETs in radio-frequency identification transponders^20,65–67^.

### Conclusion

A polymeric monolayer of PffBT4T-2DT has been fabricated by tuning polymer pre-aggregation in solution and using controllable processing technique. The monolayer exhibits strong edge-on orientation, evidenced by direct GIWAXS measurement. The pronounced molecular order together with contact engineering results in the achievement of high performance PoM-FETs with record mobility of 3 cm^2^ V^−1^ s^−1^ and reasonable operating conditions (*V*_T_ = 6.5 V and *I*_on_/*I*_off_ = 10^7^). The PoM-FET performance parameters are identical to the bulk film. The performance uniformity, large current modulation and high reproducibility of the PffBT4T-2DT PoM-FETs allowed the high-level integration of PoM-FETs into inverters to form unipolar logic gates. Ring oscillators are realized due to the small parameter spread of the individual inverters. Demonstration of a 15-bit code generator bridges the gap between discrete transistors and an integrated circuit based on a polymer monolayer, which is a big leap forward for bottom-up fabrication of plastic electronics.

## Methods

### Materials

PffBT4T-2DT was synthesized according to the literature procedure^42^. The molecular weight (*M*_n_) is 47.3 kg mol^−1^ with *M*_w_/*M*_n_ of 1.7 achieved by gel permeation chromatography (GPC) at 135 °C in 1,2,4-trichlorobenzene.

### UV–Vis absorption

UV–Vis absorption spectra were recorded by using a PerkinElmer Lambda 9 spectrophotometer at room temperature for all samples. The polymer solution in chloroform at 0.025 mg mL^−1^ was prepared, and polymer mono- and multilayers were fabricated on the quartz substrates by dip-coating from a 0.5 mg mL^−1^ chloroform solution.

### GIWAXS characterization

All samples were prepared on Si substrates using identical solutions as those used in devices, either with or without pre-patterned, PFBT treated Au electrodes. Two sets of GIWAXS measurements were performed. One set was measured from four samples with electrodes (Fig. 2a and Supplementary Fig. 4) and 1 without. The 1D profiles from a sample without electrodes are shown in comparison to a sample with electrodes at same coating speed in Supplementary Fig. 6 which shows that the electrodes significantly increased the low q scattering background but did not noticeably affect the ordering of the polymer. The aforementioned data were measured at beamline 11-3 at the Stanford Synchrotron Radiation Lightsource (SSRL) using 12.7 keV X-rays and a Rayonix MX225 CCD detector. Scattering patterns measured at this beamline have a significantly lower scattering background than the other data set (Supplementary Fig. 5, measured at beamline 7.3.3^68^ at the Advanced Light Source using 10 keV X-rays and a Dectris Pilatus 2 M photon counting detector) due to a fully helium-purged sample environment. The lower background allowed for more accurate quantitative analysis of the in-plane *π*–*π* stacking, but the scattering features in the two sets are in good qualitative agreement. The X-ray beam was incident at a grazing angle of around 0.14° for both data sets, above the critical angle of the polymer film but below that of the substrate. The data reduction from 2D to 1D profiles was performed using the NIKA^69^ software package in Igor Pro. Line profiles were calculated by averaging ±10° cake slices about a given direction using appropriate masking. Coherence lengths were calculated from peak fits using the Scherrer eq. (1):

1 $$L_{\rm C} = \frac{{2\pi }}{{\Delta q}},$$

where Δ*q* is the full width at half maximum of the peak.

### AFM characterization

The morphologies of polymer mono- and multilayers were characterized by a Dimension Icon FS AFM. The tapping mode was utilized. All samples were dip-coated on SiO_2_.

### OFET fabrication and testing

The substrates for OFET fabrication were purchased from BASF and Philips in which heavily doped silicon was used as gate electrodes, 250-nm-thick thermally grown SiO_2_ as dielectric layer and 50-nm-thick Au as source and drain electrodes. Before the process of dip-coating, these substrates were firstly cleaned by 10 min ultrasonication in acetone and subsequent 10 min ultrasonication in isopropyl alcohol. Then the substrates were dried in a nitrogen flow. After activation by using argon plasma for 1 min, the Au electrodes were functionalized with self-assembled monolayers (SAMs) by immersing the cleaned substrates into a 10 mM 2,3,4,5,6-pentafluorothiophenol (PFBT, Aldrich) solution in ethanol for 6 h. Then the substrates were rinsed with ethanol and dried in a nitrogen flow. Polymer mono- and multilayers were deposited by dip-coating from a 0.5 mg mL^−1^ chloroform solution with the speeds ranging from 1000 to 50 μm s^−1^. Chloroform was evaporated during dip-coating. Therefore, a chloroform solution of 8 mL at 0.5 mg mL^−1^ was prepared in a 10-mL vial in order to keep the solution concentration almost constant. After film deposition, polymer mono- and multilayers were annealed at 100 °C for 0.5 h in a glovebox under a nitrogen atmosphere to remove the residual solvent. A Keithley 4200-SCS was used for all electrical measurements in a glovebox.

### Integrated circuits

Details of inverters, ring oscillator and 15-bit code generators are described in Supplementary Fig. 17,18,20.

### Data availability

All relevant data in this study are available from the corresponding authors upon request.

## Electronic supplementary material

Supplementary Information
