# Supplementary material for: Integrated circuits based on conjugated polymer monolayer
Source: Nat Commun. 2018 Jan 31;9:451. doi: 10.1038/s41467-017-02805-5 (PMC5792516; doi:10.1038/s41467-017-02805-5)
Supplement: Supplementary file 1 — Supplementary Information [file 41467_2017_2805_MOESM1_ESM.pdf]

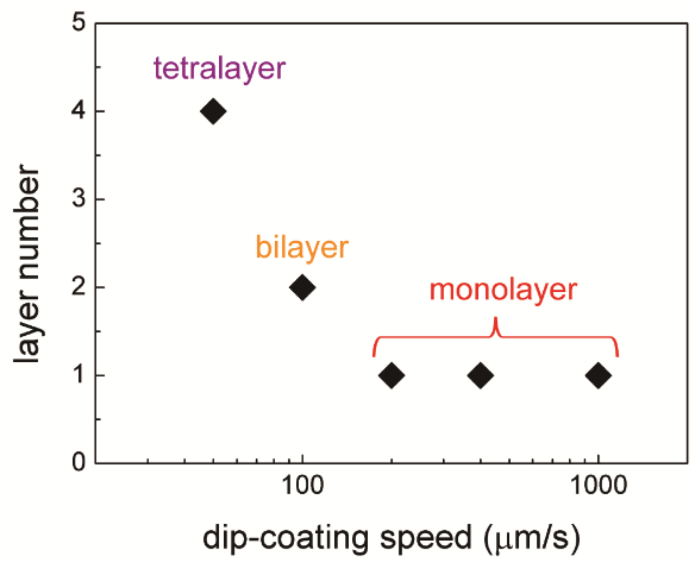

Supplementary Figure 1. Polymer layer number as a function of dip-coating speed.

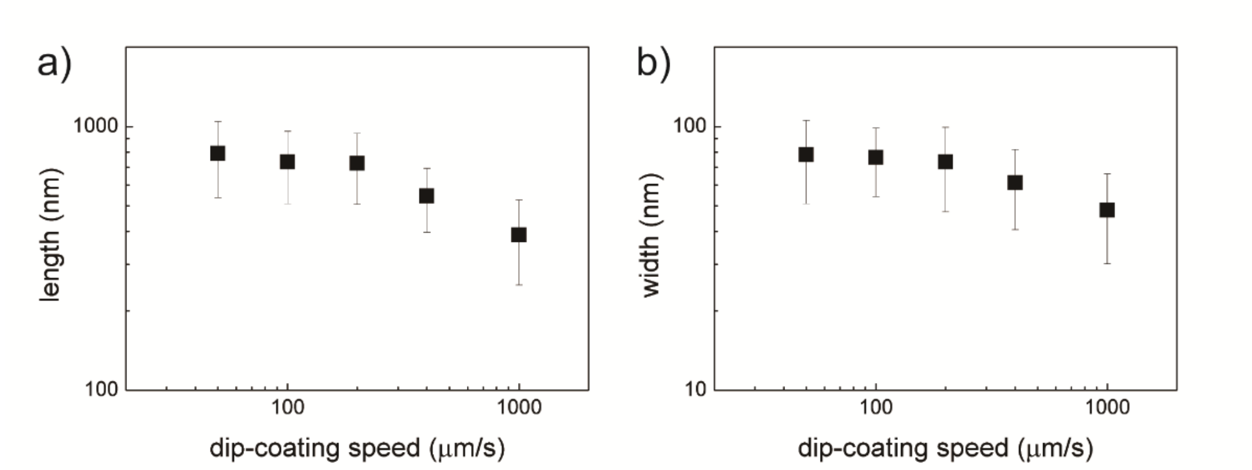

Supplementary Figure 2. The analysis of fiber dimensions. The average value and error bar (standard deviation) for each sample are analyzed from over 100 fibers by Gwyddion software.

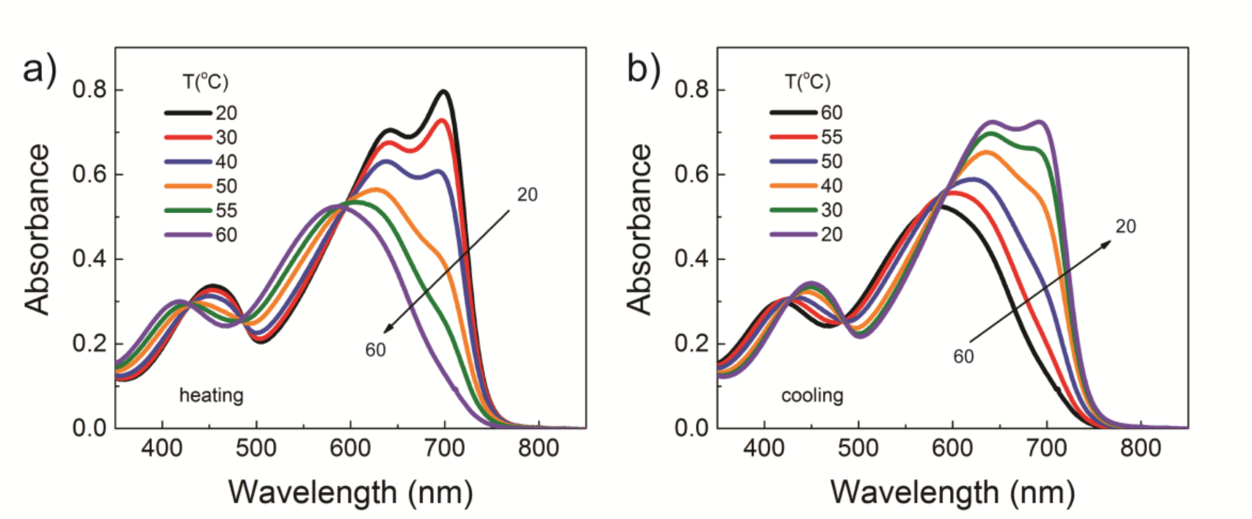

Supplementary Figure 3. Temperature-dependent absorption spectra of PffBT4T-2DT chloroform solution with polymer concentration of 0.025 mg mL^{-1}.

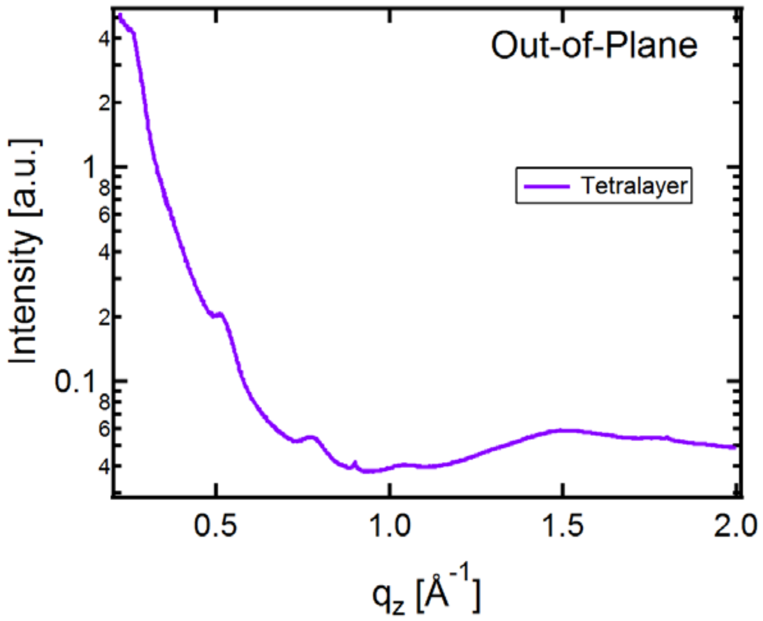

Supplementary Figure 4. Out-of-plane GIWAXS line profile of tetralayer.

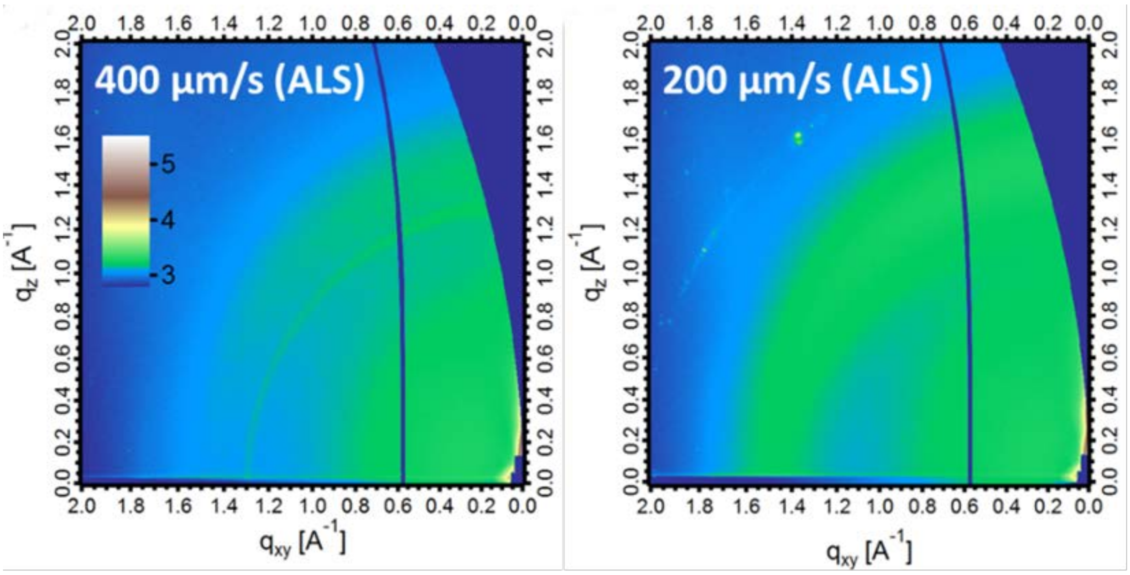

Supplementary Figure 5. 2D GIWAXS patterns of PffBT4T-2DT monolayers deposited from 400 and 200 μm s^{-1} at a different beamline (with a larger scattering background; ALS).

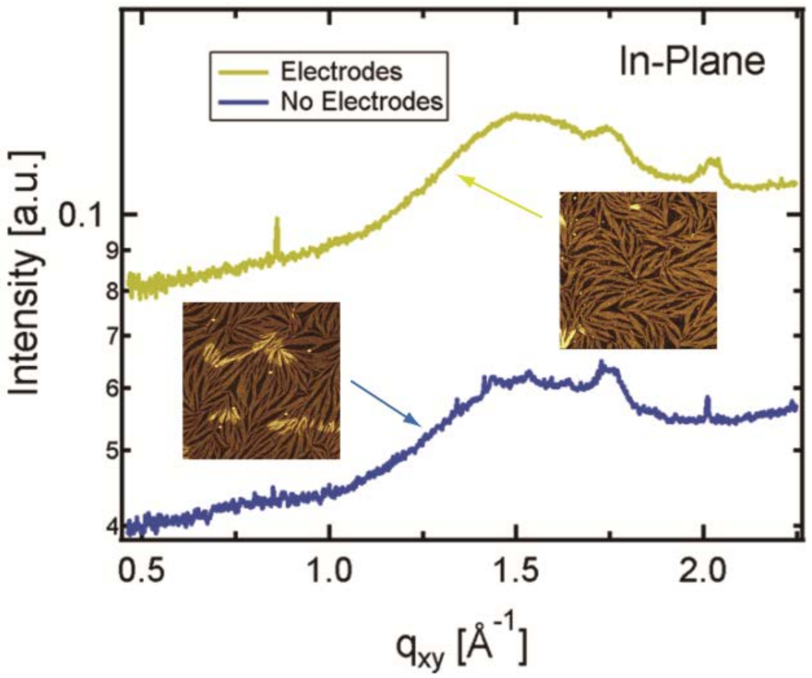

Supplementary Figure 6. In-plane GIWAXS line profiles of PffBT4T-2DT bilayers dip-coated on the substrates with and without pre-patterned electrodes. The dip-coating speed is 100 μm s^{-1}. Little difference between scattering features from the two samples can be observed. The scattering background is higher for the sample with electrodes, particularly at low q, where the difference is almost an order of magnitude. Insets are the corresponding AFM height images showing similar fibrillary morphologies for both cases.

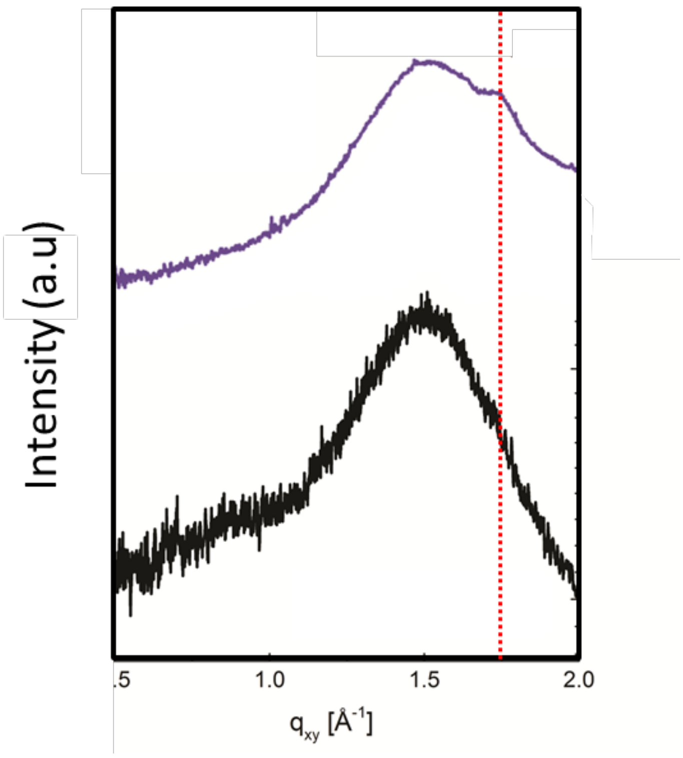

Supplementary Figure 7. GIWAXS in-plane profiles of PffBT4T-2DT tetralayers with high (top, this study, M_n = 47.3 kg mol^{-1}) and low molecular weight (bottom, Ref. 14, M_n = 23.2 kg mol^{-1}). Red dotted line indicates the peak position at q = 1.75 Å^{-1}, where π–π stacking is clear for high molecular weight (top) but disappears for low molecular weight (bottom).

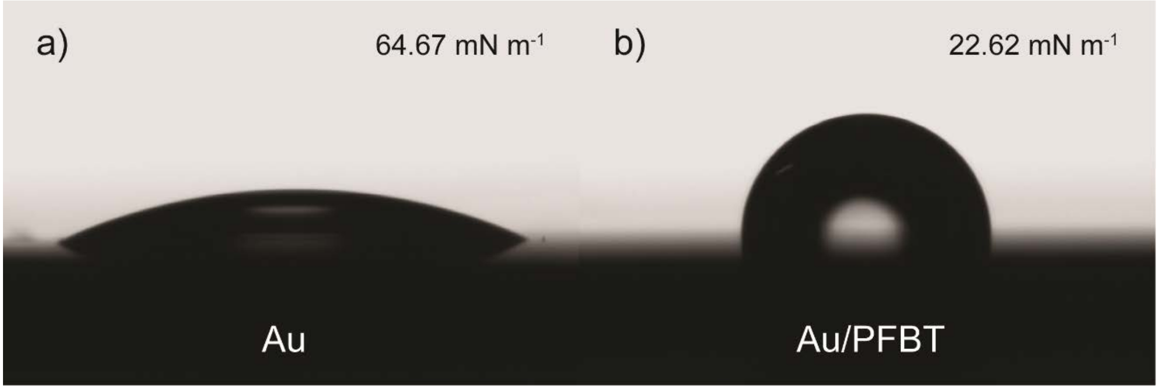

Supplementary Figure 8. Contact angle measurement for the Au surface (a) without and (b) with PFBT SAM modification. 3 μL of water was used for measurement. The contact angle is 29.6^{o} and 100.7^{o} for a,b) respectively, and the resultant surface tension is 64.67 mN m^{-1} and 22.62 mN m^{-1} by using Neumans method[15].

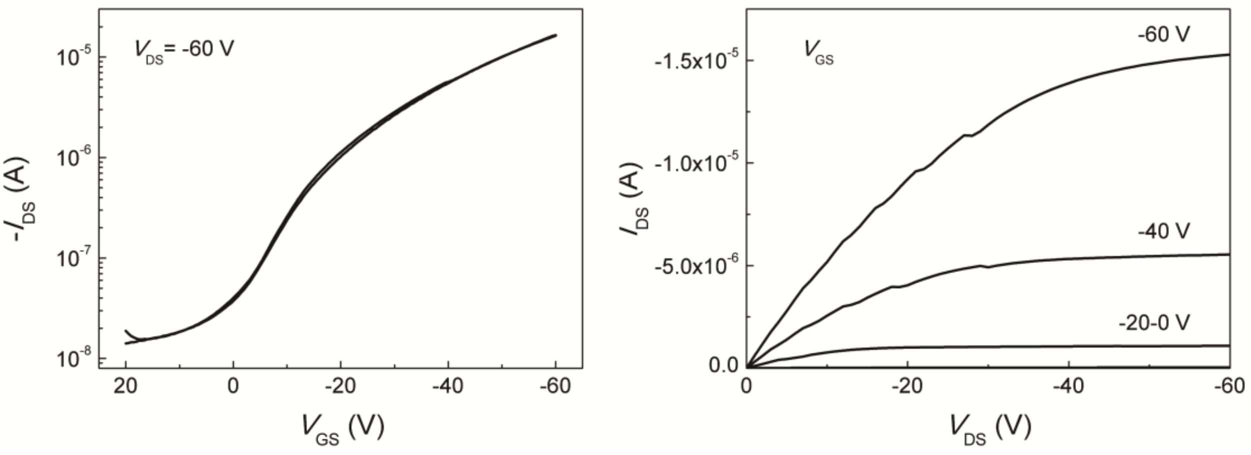

Supplementary Figure 9. Transfer and output characteristics of PffBT4T-2DT monolayer transistor in TCBG geometry. The highest field-effect mobility of the PoM-FETs obtained in TCBG amounted to a 0.1 $cm^2\ V^{-1}\ s^{-1}$.

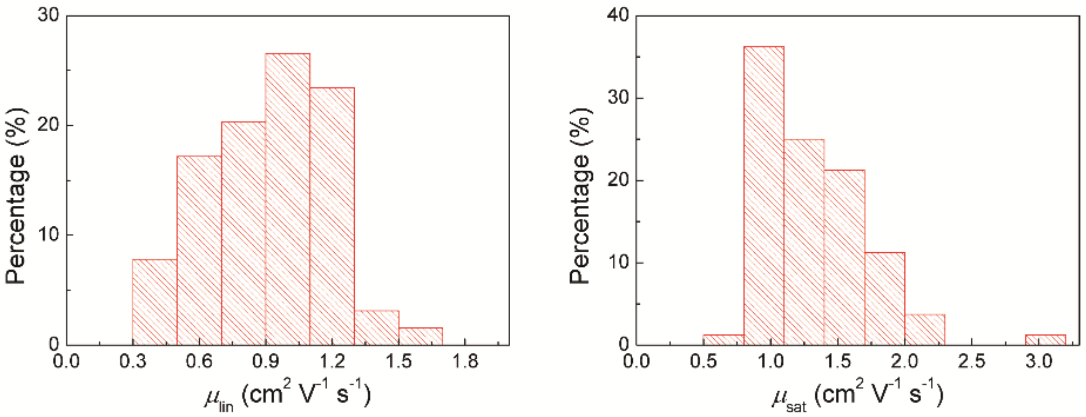

Supplementary Figure 10. The distribution of saturation and linear mobility (μ_{sat} and μ_{lin}) of PffBT4T-2DT monolayer transistor. Over 80 transistors were measured.

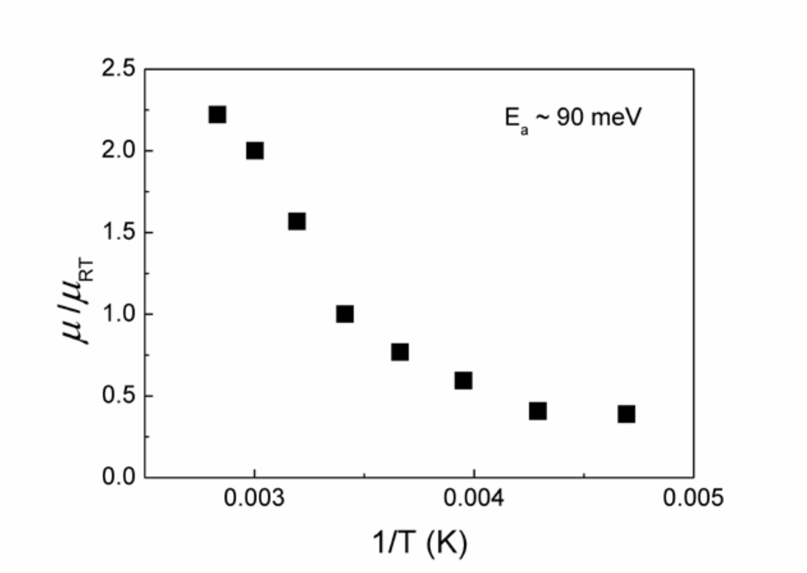

Supplementary Figure 11. The saturation mobility of PffBT4T-2DT monolayer as a function of temperature. μ is the saturation mobility measured at different temperatures, and μ_{RT} is the value measured at room temperature. The activation energy is around 90 meV.

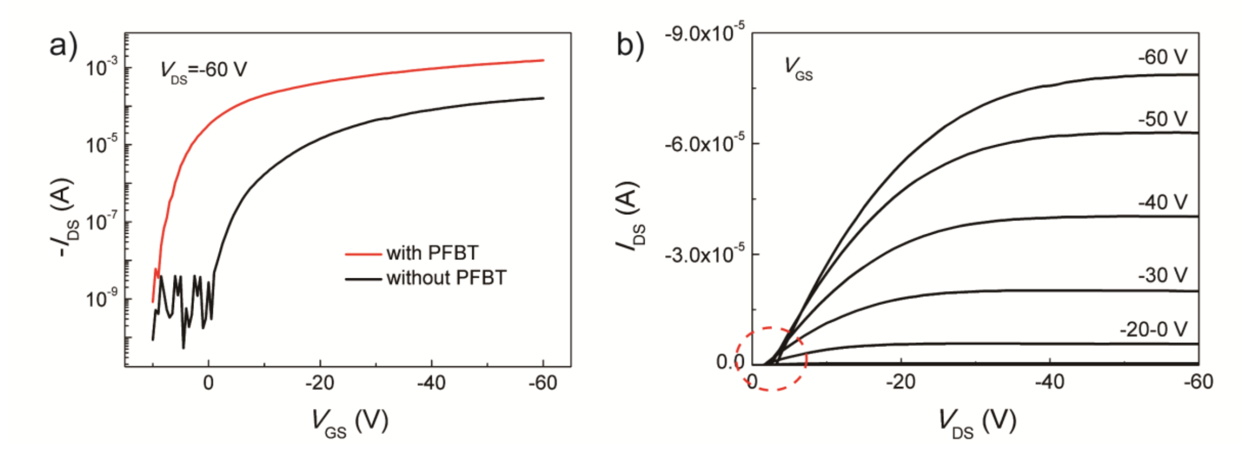

Supplementary Figure 12. a) Transfer characteristics of PffBT4T-2DT PoM-FET with and without PFBT SAM modification for Au electrodes. The channel length and width are 100 and 7000 μm, respectively. b) Output characteristics of PffBT4T-2DT PoM-FET without electrode modification. The drain current at low V_{DS} highlighted by red dash circle indicates contact resistance between polymer monolayer and Au electrodes.

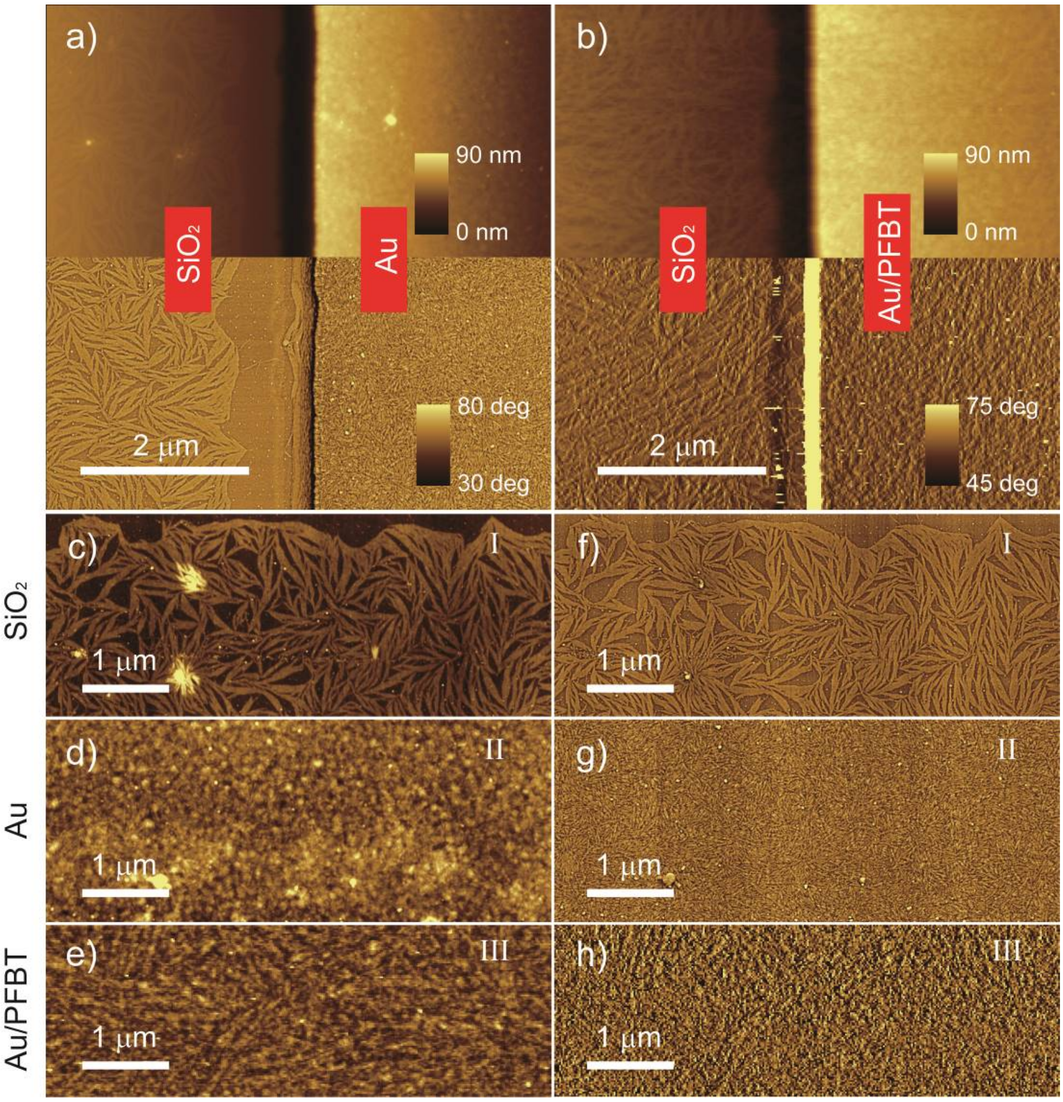

Supplementary Figure 13. a, b) AFM height (top) and phase (bottom) images of PffBT4T-2DT monolayer on the contact without and with PFBT SAM modification. c-e) Enlarged AFM height images of monolayer on SiO_2, Au electrode and PFBT-modified electrode, respectively. f-h) Corresponding phase images of c-e).

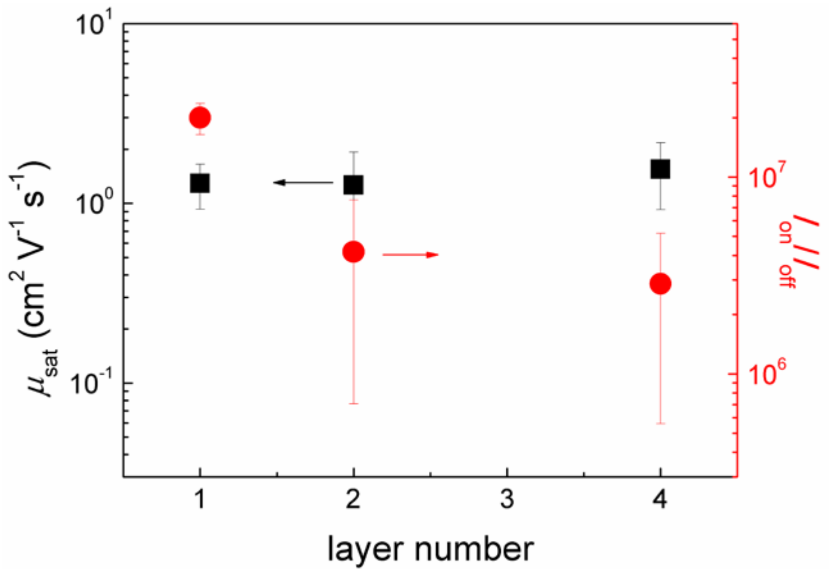

Supplementary Figure 14. The saturation mobility and on/off ratio as a function of layer number forPffBT4T-2DT PoM-FET. It is evident that the charge carrier transport remains independent of layer number. There is no obvious difference in the molecular order between films of various thickness as determined by GIWAXS (Figure 2a). The identical transistor performance of monolayer and multilayers provides further evidence that the first monolayer close to the dielectric is mainly responsible for the charge carrier transport.[16]

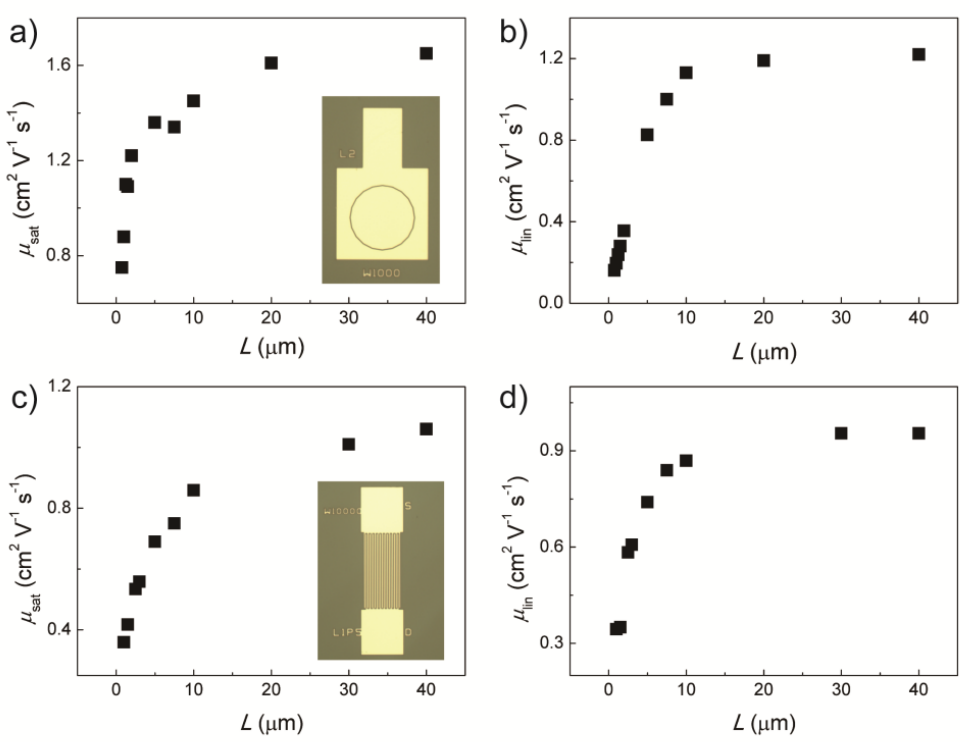

Supplementary Figure 15. The influence of channel length on the saturation (a,c) and linear mobilities (b,d) of PffBT4T-2DT PoM-FET. Both ring (a,b) and interdigitate transistors (c,d) are analyzed. The channel width is 1000 and 10000 μm for ring and interdigitate transistors, respectively. The insets in a,c) are the optical images of ring and interdigitate transistors.

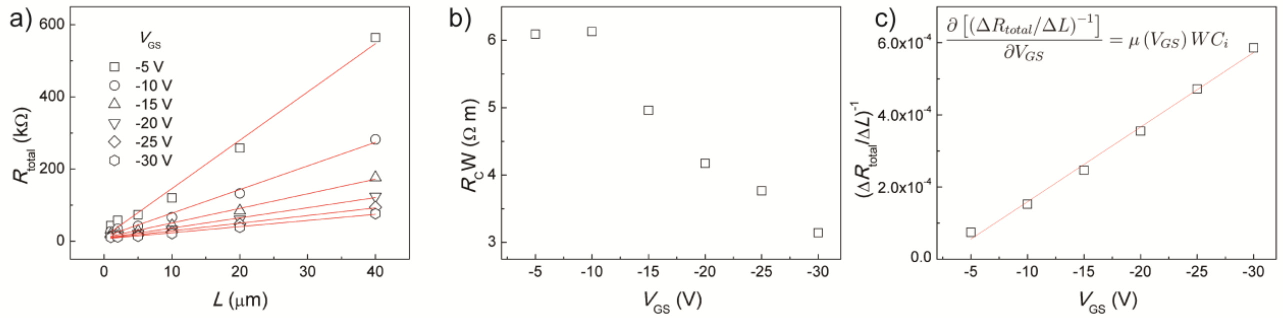

Supplementary Figure 16. a) Total device resistance (R_{total}) as a function of the channel length (L) with various V_{GS} for PffBT4T-2DT monolayer transistor. The channel width is 1000 μm. b) Contact resistance times channel width (W) as a function of V_{GS}. c) The slopes of red lines in a), $(\Delta R_{total}/\Delta L)^{-1}$, as a function of V_{GS}. According to the inset equation, the mobility calculated by transfer-length-method is 1.3 $cm^2\ V^{-1}\ s^{-1}$, in an excellent agreement with the value extruded from the transfer characteristics.

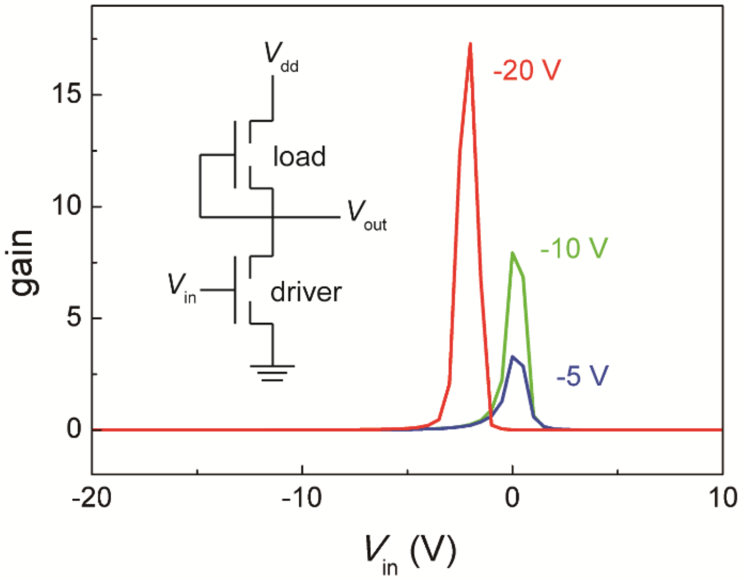

Supplementary Figure 17. Plots of corresponding gain of polymer monolayer inverter (Figure 4a). V_{in}, input voltage; V_{out}, output voltage. The inset shows a diagram of the logic gate.

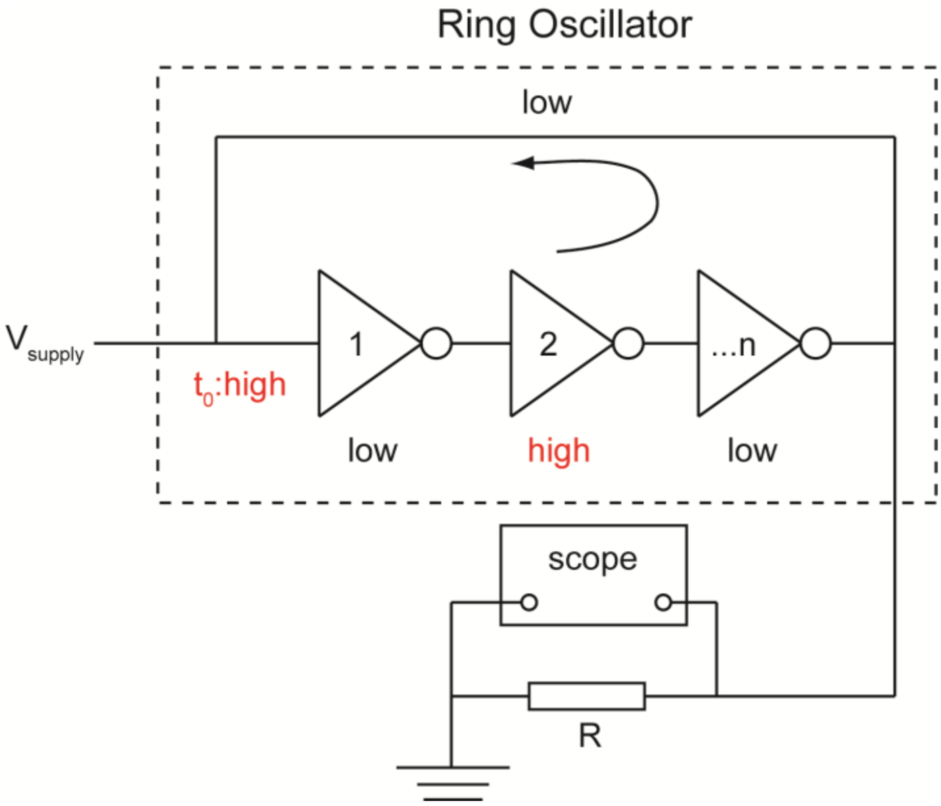

Supplementary Figure 18. Schematic illustration of the measurement set-up for the ring oscillator.

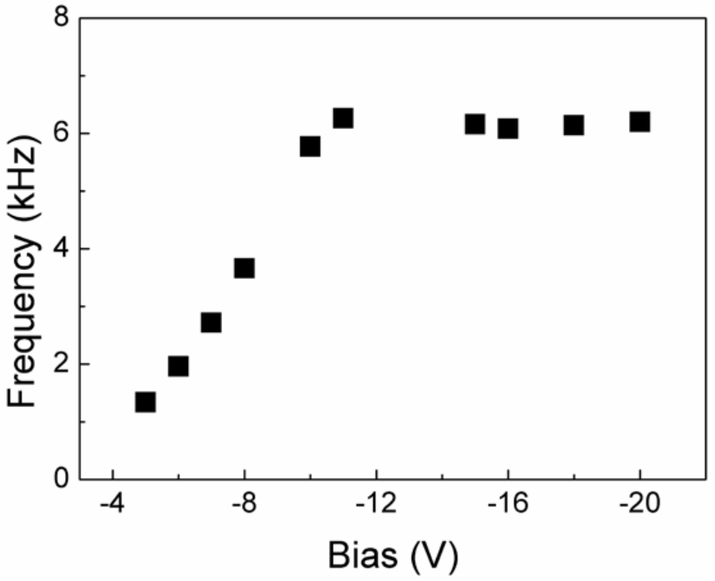

Supplementary Figure 19. The frequency of polymer monolayer ring oscillator as a function of bias voltage.

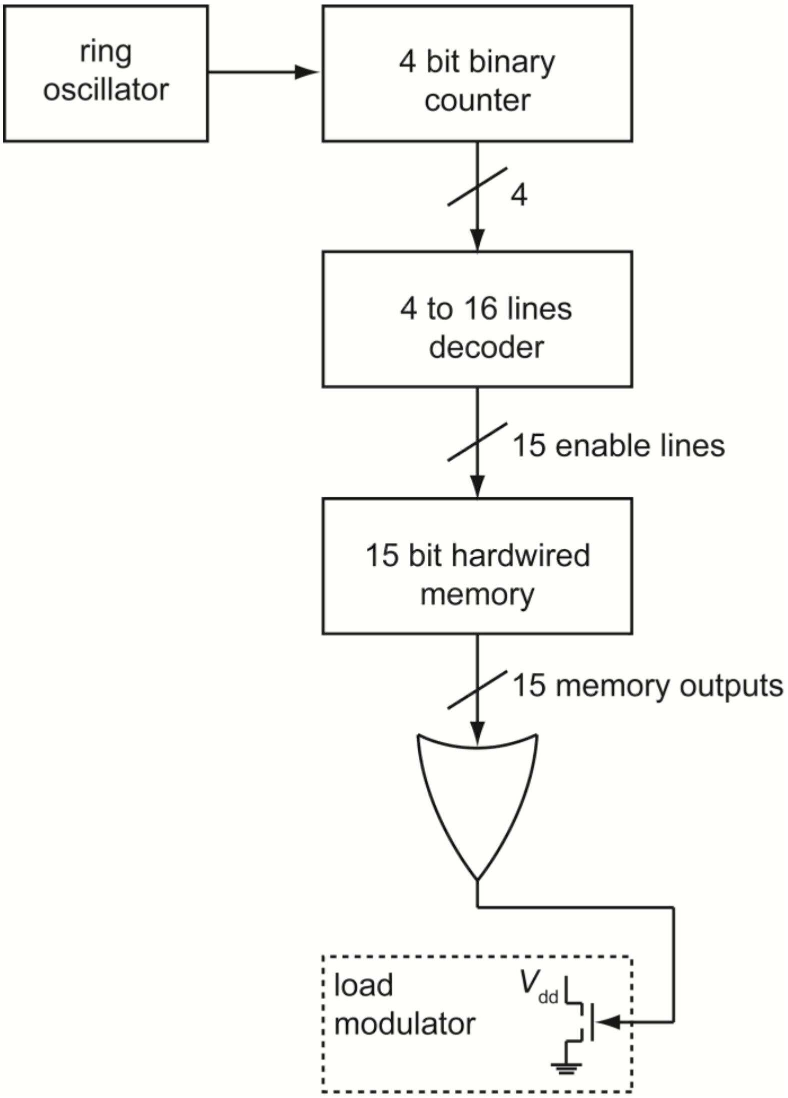

Supplementary Figure 20. Block diagram of the 15-bit code generator.

Supplementary Table 1. Summary of PoM-FETs reported in literature and this work.

Polymers	Processing technology	Morphology	Thickness (nm)	μ_h ($cm^2V^{-1}s^{-1}$)^a	V_T (V)	I_{on}/I_{off}	Circuit Integration			Year	Ref
							inverter	oscillator	code generator		
P3HT/AA	Langmuir-Blodgett	film	3.7	10^{-9}	-	-	×	×	×	1990	1
P3HT	Langmuir-Blodgett	film	-	$2×10^{-2}$	-	-	×	×	×	2000	2
P3HT	Dip-coating	film	2	$5.2×10^{-5}$	-	10^3	×	×	×	2002	3
PDA	Langmuir-Blodgett	film	1.6	-	-	-	×	×	×	2006	4
P3BT	Spin-coating	Nanofiber networks	3-4	$1.7×10^{-2}$	-10.4	$5×10^4$	×	×	×	2010	5
		Isolated nanofiber		$4.9×10^{-2}$	-15.6	$2×10^3$	×	×	×		
P3HT	Spin-coating	Nanofiber networks	3-4	$1.1×10^{-2}$	-5.3	$5×10^4$	×	×	×	2010	5
		Isolated nanofiber		$6.4×10^{-2}$	-11.3	$4×10^3$	×	×	×		
P3OT	Spin-coating	Nanofiber networks	3-4	$1.4×10^{-2}$	-7.4	$8×10^4$	×	×	×	2010	5
		Isolated nanofiber		$6.1×10^{-2}$	-4.8	$8×10^3$	×	×	×		
P3DT	Spin-coating	Nanofiber networks	3-4	$1.1×10^{-2}$	-6.7	$4×10^4$	×	×	×	2010	5
		Isolated nanofiber		$3.3×10^{-2}$	-3.7	$6×10^3$	×	×	×		
P3HT	Langmuir-Blodgett	film	-	$1.0×10^{-3}$	-	10^5	×	×	×	2010	6
P(NDI2OD-T2)	Langmuir-Schäfer	film	3.1	$2×10^{-3b}$	35	10^3	×	×	×	2012	7
P3HT	Spin-coating	film	2	$1.7×10^{-5}$	5.4	-	×	×	×	2013	8
P3HT	Aged solution	nanowhiskers	1.3	$1.3×10^{-2}$	-	-	×	×	×	2013	9
P3HT	Spin-coating	film	2.5	$5×10^{-2}$	-	-	×	×	×	2014	10
P(NDI2OD-T2)	Bar-coating	Nanofiber network	2.2	0.14^b	-	10^5	×	×	×	2015	11
DPPT-TT	Bar-coating	Nanofiber network	2.2	1.1	-46.5	10^7	×	×	×	2016	12
P(NDI2OD-T2)	Bar-coating	Nanofiber network	1.7	0.14^b	34.7	10^6	×	×	×	2016	12
IIDDT-C3	Spin-coating	Nanofiber network	3.1	0.02	-	-	×	×	×	2017	13
	Langmuir-Schäfer	Nanofiber network	4.5	0.04	-	-	×	×	×	2017	
PffBT4T-2DT	Dip-coating	Nanofiber network	2.4	3.02	-6.5	10^7	√	√	√	2017	This work

[a] Saturated mobility for holes; [b] saturated mobility for electrons.

Supplementary Table 2. Device performances of PffBT4T-2DT PoM-FETs with three different geometries of source/drain (S/D) patterns.

S/D patterns	μ_{sat} (cm^2 V^{-1} s^{-1})	μ_{lin} (cm^2 V^{-1} s^{-1})	V_T (V)	I_{on}/I_{off}
co-centric ring	1.39±0.48	0.99±0.32	8.7±2.5	10^7-10^8
interdigitate	1.17±0.32	0.88±0.20	8.6±2.3	10^7-10^8
linear	1.29±0.36	0.57±0.03	3.3±3.4	10^7-10^8
average[a)]	1.31±0.41	0.90±0.28	6.5±3.8	10^7-10^8

[a)] Over 80 devices were measured.

Supplementary Note 1

The PoM-FET present in this work exhibits a record field-effect mobility of 3.02 $cm^2 V^{-1} s^{-1}$ with reasonable operating conditions (V_T = 6.5 V and $I_{on}/I_{off} = 10^7$), which is one order of magnitude (on average) higher than the state-of-the-art in literature. More importantly, this work shows a big leap forward for bottom-up fabrication of plastic electronics by reporting the first example of an IC based on a monolayer of a high mobility conjugated polymer. In this work, we define inverter only as one of the components of the IC.

Supplementary Note 2

Neumans' equation (1) was used to calculate the surface tension:

$$(\frac{\gamma_s}{\gamma_l})^{0.5} e^{-\beta_1(\gamma_l-\gamma_s)^2} = 0.5(1 + cos\theta) \quad (1)$$

where θ is contact angle, γ_s and γ_l are the surface tension for solid surface (s) and liquid surface (l). β_1 = 0.0001247, and γ_l = 72.8 mN m^{-1} for water. We obtained 64.67 mN m^{-1} and 22.62 mN m^{-1} for the surface tension of Au and Au/PFBT, respectively.

Supplementary Note 3

In this work, BCBG geometry is mainly employed for two reasons. First, PoM-FET based on this geometry exhibits one order of magnitude higher mobility compared to TCBG ones (Supplementary Figure 9). Second, an IC was only possible by depositing the polymer onto pre-patterned substrate.

Supplementary Note 4

The design and fabrication of the pre-patterned substrates for integrated circuits have been done in our previous work, and the detailed information can be found in Ref. 17.

Supplementary References

1. Paloheimo, J., Kuivalainen, P., Stubb, H., Vuorimaa, E. & Yli-Lahti, P. Molecular field-effect transistors using conducting polymer Langmuir–Blodgett films. *Appl. Phys. Lett.* **56**, 1157-1159 (1990).
2. Xu, G., Bao, Z. & Groves, J. T. Langmuir−Blodgett films of regioregular poly(3-hexylthiophene) as field-effect transistors. *Langmuir* **16**, 1834-1841 (2000).
3. Sandberg, H. G. O., *et al*. Ultrathin Regioregular Poly(3-hexyl thiophene) Field-Effect Transistors. *Langmuir* **18**, 10176-10182 (2002).
4. Scott, J. C., Samuel, J. D. J., Hou, J. H., Rettner, C. T. & Miller, R. D. Monolayer transistor using a highly ordered conjugated polymer as the channel. *Nano Lett.* **6**, 2916-2919 (2006).
5. Samitsu, S., Shimomura, T., Heike, S., Hashizume, T. & Ito, K. Field-effect carrier transport in poly(3-alkylthiophene) nanofiber networks and isolated nanofibers. *Macromolecules* **43**, 7891-7894 (2010).
6. Watanabe, S.-i., *et al*. Electron spin resonance observation of field-induced charge carriers in ultrathin-film transistors of regioregular poly(3-hexylthiophene) with controlled in-plane chain orientation. *Appl. Phys. Lett.* **96**, 173302 (2010).
7. Fabiano, S., *et al*. From monolayer to multilayer n-channel polymeric field-effect transistors with precise conformational order. *Adv. Mater.* **24**, 951-956 (2012).
8. Park, B., Aiyar, A., Hong, J.-i. & Reichmanis, E. Electrical contact properties between the accumulation layer and metal electrodes in ultrathin poly(3-hexylthiophene)(P3HT) field effect transistors. *ACS Appl. Mater. Interfaces* **3**, 1574-1580 (2011).
9. Guo, Y., Jiang, L., Ma, X., Hu, W. & Su, Z. Poly(3-hexylthiophene) monolayer nanowhiskers. *Polym. Chem.* **4**, 4308-4311 (2013).
10. Xu, Z.-X. & Roy, V. A. L. Charge transport in monolayer poly(3-hexylthiophene) thin-film transistors. *Chin. Phys. B* **23**, 048501 (2014).
11. Bucella, S. G., *et al*. Macroscopic and high-throughput printing of aligned nanostructured polymer semiconductors for MHz large-area electronics. *Nat. Commun.* **6**, 8394 (2015).
12. Khim, D., Ryu, G.-S., Park, W.-T., Kim, H., Lee, M. & Noh, Y.-Y. Precisely controlled ultrathin conjugated polymer films for large area transparent transistors and highly sensitive chemical sensors. *Adv. Mater.* **28**, 2752-2759 (2016).
13. Bonacchi, S., *et al*. High, anisotropic, and substrate-independent mobility in polymer field-effect transistors based on preassembled semiconducting nanofibrils. *ACS Nano* **11**, 2000-2007 (2017).
14. Li, M., An, C., Marszalek, T., Baumgarten, M., Müllen, K. & Pisula, W. Impact of interfacial microstructure on charge carrier transport in solution-processed conjugated polymer field-effect transistors. *Adv. Mater.* **28**, 2245-2252 (2016).
15. Zenkiewicz, M. Methods for the calculation of surface free energy of solids. *J. Achiev. Mater. Manuf. Eng.* **24**, 137-145 (2007).
16. Dinelli, F., Murgia, M., Levy, P., Cavallini, M., Biscarini, F. & de Leeuw, D. M. Spatially correlated charge transport in organic thin film transistors. *Phys. Rev. Lett.* **92**, 116802 (2004).
17. Smits, E. C. P., *et al*. Bottom-up organic integrated circuits. *Nature* **455**, 956-959 (2008).
